# Post-cardiac Surgery Pleural Effusion and the Overlooked Importance of the Diaphragm: Setting Up an Inspiratory Training Program Correctly

**DOI:** 10.7759/cureus.114779

**Published:** 2026-08-19

**Authors:** Bruno Bordoni, Bruno Morabito, Allan R Escher

**Affiliations:** 1 Physical Medicine and Rehabilitation, Foundation Don Carlo Gnocchi, Milan, ITA; 2 Physical Medicine and Rehabilitation, Associazione Italiana di Riabilitazione Cardiovascolare (AIRC), Milan, ITA; 3 Physical Medicine and Rehabilitation, School of Osteopathic Centre for Research and Studies, Milan, ITA; 4 Oncologic Sciences, University of South Florida Morsani College of Medicine, Tampa, USA; 5 Anesthesiology/Pain Medicine, H. Lee Moffitt Cancer Center and Research Institute, Tampa, USA

**Keywords:** airc, cardiac rehabilitation, cardiovascular disease, coronary artery bypass grafting, diaphragm, inspiratory muscle training, physiotherapy, pleural effusion

## Abstract

Pleural effusion (PE) is one of the most common complications after cardiac surgery. PE can cause both functional and structural changes in the diaphragm, leading to longer hospital stays and higher mortality rates. This narrative review article proposes a hypothesis regarding the inspiratory muscle training (IMT) parameters that patients could achieve in various clinical settings. We selected review articles and excluded case reports, editorials, and letters. We reviewed the most significant and recent articles on PubMed. This article defines PE, examines possible mechanisms of its onset after cardiac surgery, and identifies potential risk factors. It explores how the thorax and diaphragm adapt, emphasising the need to direct clinical attention to this muscle and to IMT, not only to promote the resolution of PE but also to improve the patient's symptoms. Diaphragm rehabilitation is a strategic measure to counteract PE. Based on the literature reviewed in the article, the Italian Association of Cardiovascular Rehabilitation (AIRC) proposes, as a hypothesis to be evaluated in future research, new IMT parameters: for red fibers 30% of PImax, 2-3 sets of 15 inhalations, with a 90 second rest between one set and the next and for the white fibres: 50% of PImax, 2-3 sets of 8-12 inhalations, with a three-minute rest between sets, 2-3 days a week. IMT, already present in the literature with parameters from different authors, is a fundamental strategy for improving the clinical picture of patients with PE.

## Introduction and background

Cardiovascular disease (CVD) remains the leading cause of death and illness worldwide, including ischemic heart disease (IHD); CVD-related fatalities are estimated to reach about 18 million people annually [1-3]. On a global scale, CVD affected 626 million individuals in 2023, compared to 311 million in the 1990s, showing a rising trend [1]. IHD caused nine million deaths, with a higher percentage in the Oceania region compared to the lowest percentage in the Asia-Pacific region [1]. The European region had a CVD prevalence of 7.8% of the population in 2021, approximately 10.1% in the Eastern Mediterranean region, 7.9% in the African region, and 7.1% and 6.7% in South-East Asia and the Western Pacific, respectively [1]. The Global Burden of Disease (GBD) reports that in 2021, the Americas region experienced two million deaths from CVD, with 43 million patients with related morbidity, and the number of cases increasing from nearly three million in the 1990s to 4.4 million patients in 2021 [2].

One of the elective options for patients with CVD, following clinical assessment, is cardiac surgery, with an estimated 1-1.5 million procedures performed annually worldwide [3]. Coronary artery bypass grafting (CABG) is the most common procedure performed in Europe, America, and parts of Asia to improve survival in CVD, with valve replacement or repair as the next most frequent interventions [3-6]. In the United States, 150, 000 CABG surgeries were recorded in 2021, and 98, 504 aortic valve replacement surgeries (mainly for stenosis or calcific valves) were documented in 2022 [2,5-7]. Globally, CABG accounts for approximately 70% of all cardiac surgical procedures, with a mortality rate of 2.5%; the mortality rate for aortic valve replacement is 2.3%, rising to 4.4% if the patient also undergoes CABG, and to 10% with concomitant mitral valve replacement [8]. The mortality rate for mitral valve replacement ranges from 1.1% to 5.5%, whether performed alone or in combination with other procedures [8].

One of the complications following cardiac surgery is pleural effusion (PE), which occurs in 43-91% of cases. PE can be detected in the days after surgery but also several months later, accompanied by possible dyspnea at rest and during exertion, as well as pleural pain at the site of the effusion [9]. PE may cause functional alterations of the diaphragm, potentially leading to longer hospital stays and higher mortality [10]. Currently, there is no gold standard to prevent the onset of PE. This article reviews the definition of PE, possible mechanisms of its development after cardiac surgery, and associated risk factors. It examines how the thorax and diaphragm adapt to the presence of effusion, emphasising the importance of focusing clinical attention on this muscle, not only to promote PE resolution but also to improve patient symptoms. This narrative review article proposes a hypothesis regarding the inspiratory muscle training (IMT) parameters that patients could achieve in various clinical settings. This article represents the position paper of the Italian Association of Cardiovascular Rehabilitation (AIRC). The hypotheses presented need to be further explored and validated through additional research. Currently, there is no validated and well-structured protocol on IMT and PE in the scientific literature.

## Review

Selection criteria

The search timeframe is from 1999 to 2026. The database searched is PubMed

The principal search terms or conceptual combinations used are pleural effusion; pleural effusion and diaphragm; inspiratory muscle training; inspiratory muscle training and pleural effusion; cardiovascular disease and diaphragm; physiotherapy and pleural effusion; cardiac rehabilitation and pleural effusion.

The rationale for selecting included references is review articles (regardless of methodology), guidelines for the management and classification of PE, and experimental articles (preferably using human models) in the fields of physiology (to explain the presence of PE) and rehabilitation (to combat or resolve PE); articles with the largest number of patients involved in the PE setting to better frame the epidemiology and, ideally, with a broader follow-up were sought.

Pleural effusion

It is estimated that PE affects 24 million people worldwide due to various clinical causes, approximately 60 different factors, ranging from renal to cardiac, including liver cirrhosis and diabetes [11,12]. Nonmalignant pleural effusion (NMPE) in patients with chronic heart failure accounts for the highest percentage of occurrence (87%), regardless of the presence of thoracic surgery [13]. In thoracic cardiac surgery, NMPE is a common clinical event, as well as in mechanically ventilated patients (60%) post-surgery, which leads to an increased length of stay in the intensive care unit (ICU) [9,14].

The Light's criteria (1972) serve as the gold standard for classifying PE as exudative or transudative, based on specific pleural fluid parameters and pleural aspiration. Exudative versus transudative PE is indicated by a pleural fluid lactate dehydrogenase (LDH) level that is ≥ 2/3 of the upper limit of normal for serum LDH, a pleural/serum LDH ratio of ≥ 0.6, and a pleural fluid/total serum protein ratio of ≥ 0.5; additionally, a total pleural fluid protein of ≥ 3 g/dL (30 g/L), a total pleural fluid cholesterol of ≥ 60 mg/dL (1.55 mmol/L) / ≥ 43 mg/dL (1.11 mmol/L), a serum/pleural cholesterol ratio of ≥ 0.3, and a serum protein/pleural fluid protein ratio of ≤ 3.1 g/dL (31 g/L) are considered [15].

**Figure 1:**
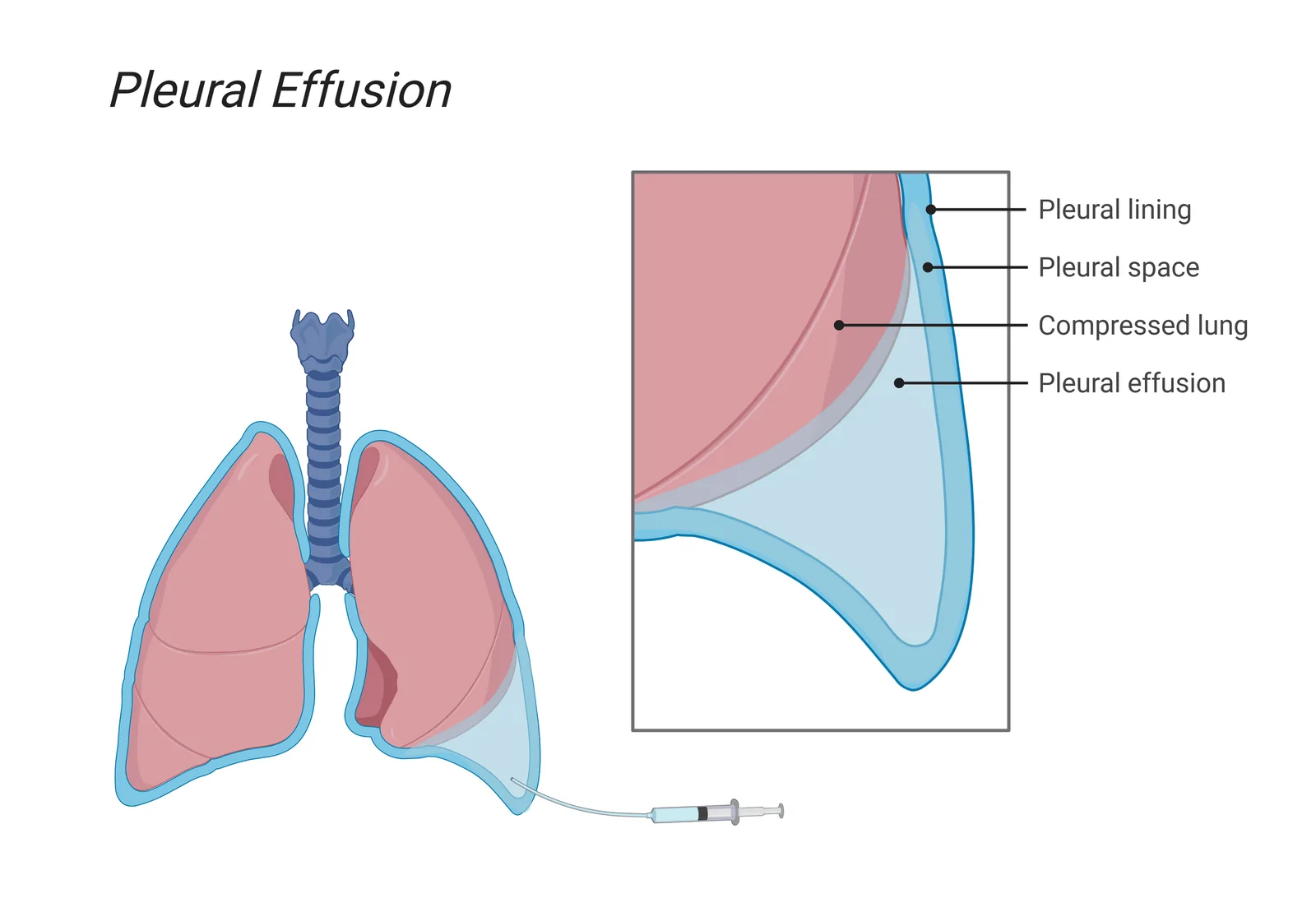
The image depicts a pleural effusion that obliterates the lung's costophrenic angle, compressing the lung tissue; it also shows pleural drainage. Drainage is necessary when an abnormal accumulation of pleural fluid in the pleural space exceeds the pleura's capacity for reabsorption and becomes significant. Currently, there are no established guidelines on the optimal way to perform a thoracentesis. The image is the property of Bordoni Bruno with a subscription to BioRender.com.

According to Heffner’s criteria, exudative PE is diagnosed when the pleural protein level exceeds 2.9 g/dL, the pleural cholesterol level exceeds 45 mg/dL, and the pleural LDH level is greater than two-thirds of the normal serum values (Figure 1) [16].

**Figure 2:**
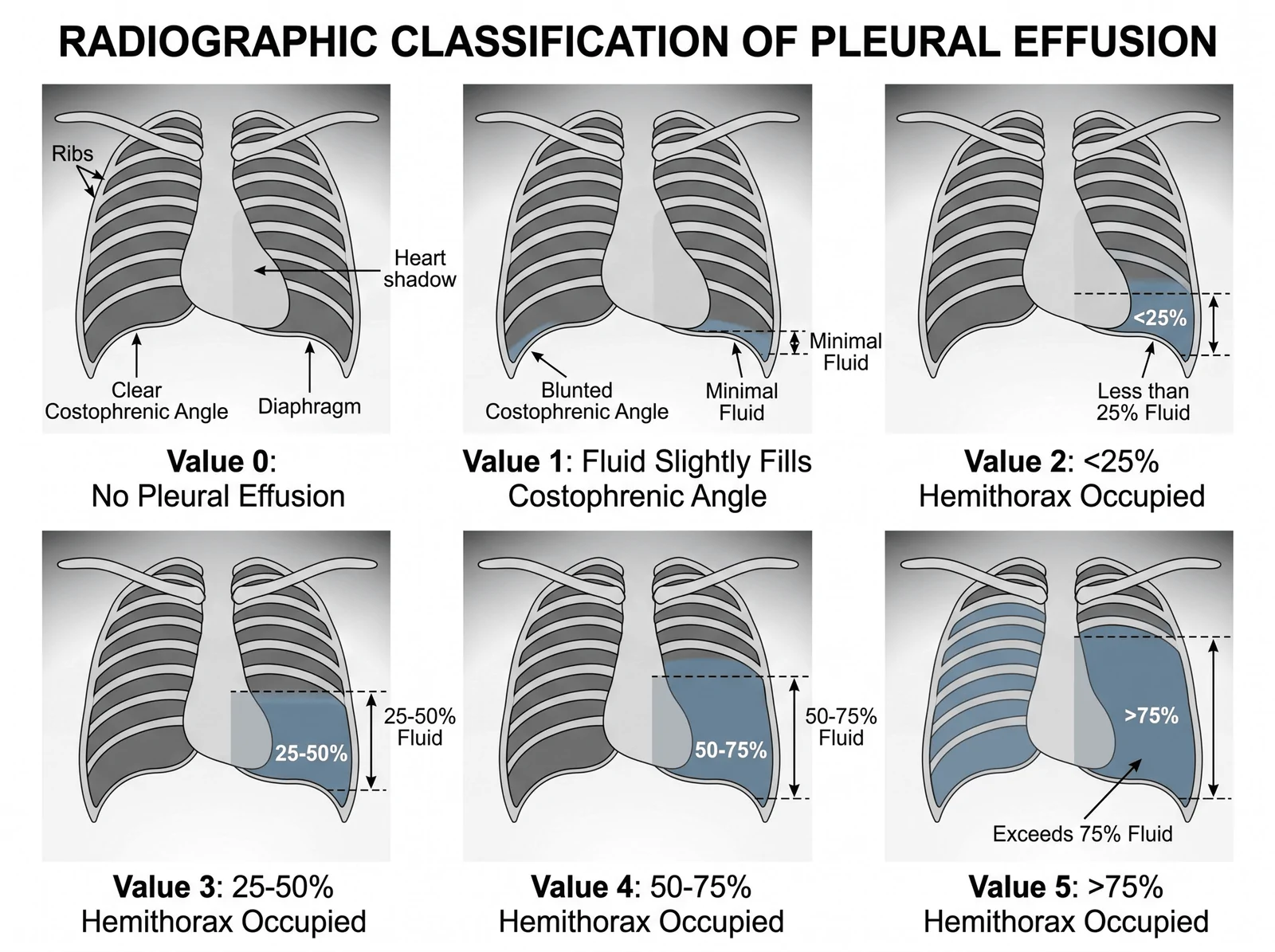
Levels of severity of pleural effusion. The image schematises the levels of severity of pleural effusion: a value of zero indicates no pleural effusion; a value of one when the costophrenic angle is slightly filled with fluid; a value of two when less than 25% of the hemithorax is occupied by fluid; a value of three when the fluid fills 25-50% of the hemithorax; a value of four if the fluid occupies 50-75% of the hemithorax; and a maximum value of five when the fluid exceeds 75% of the hemithorax. The figure with the highest pleural effusion has the contralateral (left) side coloured to emphasise the severity of the lung dysfunction; a single functioning lung will jeopardise the function of the lung not involved by the effusion. Adapted from [18] and created with Paint.net by Bruno Bordoni.

The amount of PE (radiographic examination) can be classified: a value of zero indicating no PE; a value of one when the costophrenic angle is slightly filled with fluid; a value of two when less than 25% of the hemithorax is occupied by fluid; a value of three when the fluid fills between 25-50% of the hemithorax; a value of four if the fluid occupies 50-75% of the hemithorax; and a maximum value of five when the fluid exceeds 75% of the hemithorax (Figure 2) [17].

PE may cause symptoms such as chest pain, dry cough, dyspnoea, fever, and systemic signs, regardless of whether the effusion is exudative or transudative, or the patient may have no symptoms [11,19]. Under physiological conditions, pleural fluid (0.26 mL/kg−1) is produced by blood vessels on the surface of the parietal pleura, moving due to the vessels' systemic hydrostatic pressure. The fluid is then absorbed by the lymphatic vessels present in the pleural space, which derive from the parietal pleura [19]. The lymph that collects the pleural fluid is transported towards the tracheobronchial lymphatic vessels and towards the diaphragm, which through the lymphatic vessels that are richly found in the muscle, is then carried to the Chyli cistern [20]. The movement of fluids in the parietal pleura is largely dependent on the movement of the diaphragm and the heartbeat (cyclical change in pressure), thanks to the lymphatic system [19,20]. When the amount of effusion exceeds the pulmonary drainage capacity and the lymphatic system is unable to compensate despite increased vessel diameter, fluid accumulates in the interstitial space [19]. Transudate results from an alteration in hydrostatic or oncotic pressure, while exudate results from increased microvascular permeability [21]. Increased hydrostatic pressure may result from heart failure, while decreased hydrostatic pressure may result from cardiac surgery; decreased oncotic pressure may result from renal disease [20]. PE resulting from cardiac surgery occurs due to an imbalance of Starling forces, that is, unbalanced oncotic and hydrostatic forces between the pleural space and the visceral pleura [21]. Exudate results from increased blood vessel permeability and/or impaired pleural lymphatic function due to lung disease (infection, tumour) [21].

Palpation (percussion) of the lungs may produce a dull sound, while auscultation may show a reduction in breath sounds and vocal fremitus; egophony may be heard, especially when the upper lung regions are affected [11]. PE can also be visualised by sonography (identification of PE above 90%), where it is indirectly detected as hyperechogenic echoes and anechoic lines (interpleural space), although it is challenging to determine the exact extent of the effusion as well as the nature of PE [22,23].

The differentiation between the components that make up PE (from chemical examination) does not equate to understanding the causes that lead to pleural fluid accumulation [23].

Possible mechanisms of post-cardiac surgery pleural effusion

Transudate is less common than exudate, which frequently occurs after thoracic cardiac surgery, particularly CABG [24]. What can happen during surgery that might cause PE?

Thoracic surgery, depending on the procedure type, can cause temporary hypoxia in lung tissues because blood flow through the pulmonary vascular network is severely restricted. When the obstruction to pulmonary circulation ends (arterial clamping is released), blood flow from the bronchial area decreases (within a few minutes), and this limited event worsens pulmonary ischemic phenomena [25]. This fluctuating circulatory state, involving ischemia and reperfusion, causes lung ischemia-reperfusion injury (LIRI) [26].

LIRI triggers a cascade of pro-inflammatory and pro-apoptotic substances, reduced microvessel permeability, and damage to endothelial structure, leading to pulmonary hypertension, oedema, and decreased alveolar capacity, thereby attracting neutrophils [25,26]. The cellular scaffolding of microvessels swells and tends to protrude into the vessel, causing further obstruction; the arrival of neutrophils (and platelets) further impedes blood flow, delaying the return to normal physiological conditions [25]. In the presence of LIRI, the combination of heparin and possible allogeneic transfusion promotes further neutrophil recruitment and the development of an inflamed lung environment [25].

**Figure 3:**
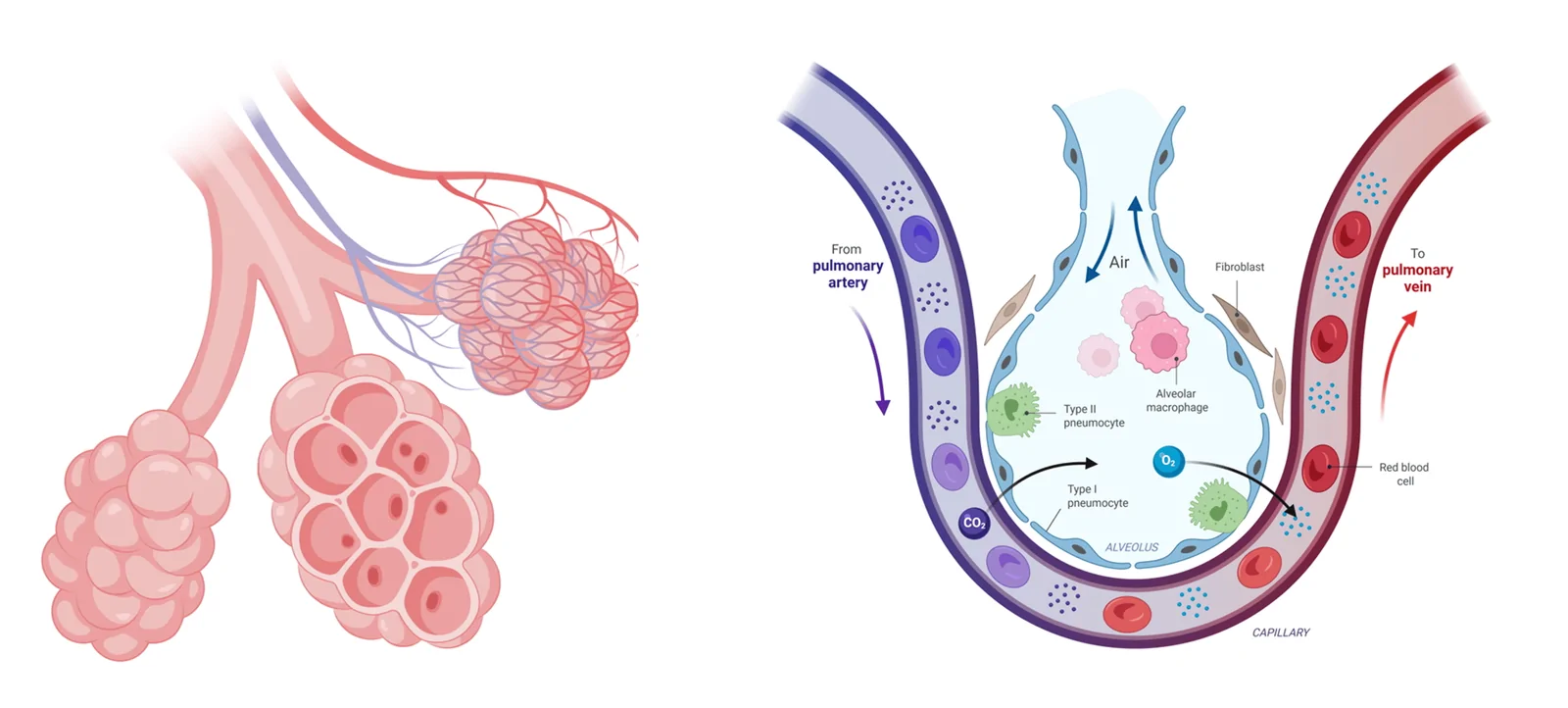
The image depicts healthy alveoli (left) encircled by a network of microvessels, illustrating physiological gas exchange between an alveolus and the microcirculation (right). Thoracic cardiac surgery can induce an inflammatory and pro-apoptotic environment within the microcirculation of the pulmonary vascular scaffold, leading to alterations in gas pathways and the subsequent build-up of pleural fluid. The image is the property of Bordoni Bruno with a subscription to BioRender.com.

The altered lung area can lead to post-surgical respiratory complications, including PE (Figure 3).

Possible risk factors for pleural effusion

In the literature, some authors highlight certain predisposing factors for the development of PE. After CABG, in particular, female gender, a low body mass index, a history of atrial fibrillation, and/or the use of anticoagulant therapy may increase the risk of developing PE [25]. CABG surgery and concomitant valve replacement may also predispose individuals to PE [25]. In patients undergoing CABG and with cardiac dysfunction, elevated N-terminal pro-B-type natriuretic peptide (NT-pro-BNP) levels (>5.000 ng/mL) are associated with an increased risk of PE [27,28].

Other factors potentially predictive of PE occurrence include the duration of mechanical intubation or ventilation after surgery and earlier removal of the surgical chest drain. Arrhythmia or fibrillation during surgery and renal dysfunction after surgery are other events that increase the rate of PE occurrence [29,30]. Other post-CABG co-variables that could more easily lead to PE include the presence of carotid stenosis, post-surgical anaemia, and longer surgery times (>4 hours) [31].

It should be remembered that PE prolongs hospital stays, with an average of 6-8 additional days, and that patients requiring secondary drainage after surgery may face an increased risk of mortality, regardless of existing comorbidities or predisposing factors [10,30]. The presence of PE also increases the risk of readmission. One to three months after cardiac surgery, PE is a significant cause of readmission; among 177,229 patients undergoing cardiac surgery, 16.7% (28,601) were readmitted, with 22.5% of these readmissions due to PE [32].

As mentioned, PE can also occur 1-3 months later, likely due to pre- or post-surgical diaphragmatic dysfunction [9,10,33].

Currently, there is no gold standard programme to prevent PE, other than early intervention after cardiac surgery, monitoring the patient's clinical progress, and, as we recommend later in the article, using non-invasive tools to stimulate the diaphragm.

Adaptation in the presence of pleural effusion

**Figure 4:**
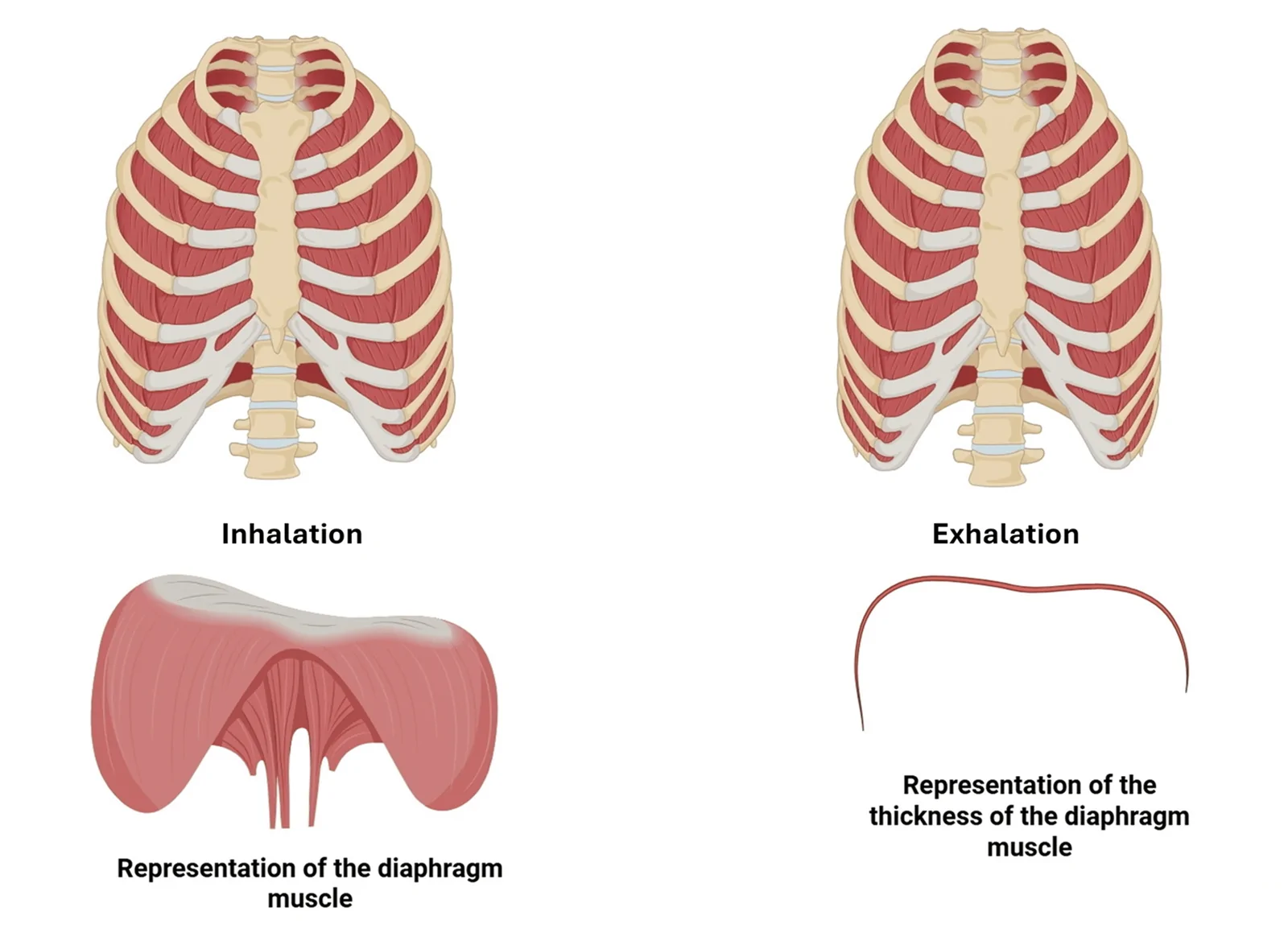
The image illustrates how the diaphragm muscle moves during inspiration and expiration. A frontal view of the diaphragm and its thinness are highlighted. During a eupneic act and through ultrasound, the diaphragm contracts approximately 13.4 ± 1.8 millimetres, while during forced inspiration it can descend about 70 ± 6 millimetres for males and roughly 57 ± 1 millimetres for women; a dysfunctional diaphragm will have a reduced range of motion by approximately 10-15 millimetres [47]. The thickness of a healthy diaphragm should be around 1.9 ± 0.4 millimetres for men and approximately 1.4 ± 0.003 millimetres for women [47]. During resting breathing in the presence of left hemilateral dysfunction, a greater movement of the right hemilateral side, about 2.8 centimetres for men and roughly 2.5 centimetres for women, can be recorded; for right hemilateral dysfunction, the left hemilateral side may move approximately 3 centimetres for men and about 2.5 centimetres for women, compared to normal values [46]. There does not seem to be a relationship between age and diaphragmatic excursion, although there appears to be a difference in the time needed to complete an inspiratory contraction, with approximately 0.5-1.7 seconds for women and around 0.4-1.8 seconds for men [48]. The image is the property of Bordoni Bruno with a subscription to BioRender.com

Dyspnoea is one of the most common symptoms in patients with PE. However, the amount of PE does not directly correlate with the presence of dyspnoea, and the causes of dyspnoea are not always clear. Not all patients experience respiratory relief after thoracentesis. [11,12,28,34,35]. Diaphragmatic dysfunction is likely a key cause of dyspnoea in the presence of PE [34]. Thoracentesis does not significantly alter forced expiratory volume in 1 second (FEV1) or oxygen saturation; the reduced lung volume seen with PE is not the primary cause of dyspnoea [11,12]. Patients who experience improvement in dyspnoea after thoracentesis tend to have larger PEs, although even in these cases, study results are inconsistent [11,36]. PE enhances chest expansion without impairing the elasticity of musculoskeletal structures, thereby reducing lung compression [11,36]. Excess pleural fluid mimics a hemiside paralysis, where the diaphragm on the affected side is pushed caudally, appearing either “flattened” on imaging or inverted due to high PE (pendulum breathing) [34,36,37]. This adaptation becomes more pronounced when supine, potentially causing dyspnea at rest [36]. The diaphragm’s position in PE reduces its apposition zone, lowering its ability to generate force or tension; it begins in a shortened state, facing biomechanical disadvantages [36]. The shortened diaphragm requires increased electrical activity from the phrenic nerve, which can lead to “neuromechanical uncoupling,” as diaphragm strength does not increase [36,38]. The unaffected hemithorax compensates with a higher breathing rate, but this may eventually cause fatigue and dyspnoea [39]. Mechanical changes in the diaphragm might stimulate phrenic afferents and other mechanoreceptors, sending abnormal signals that could cause dyspnoea and influence the respiratory centres, thereby supporting the function of the unaffected hemidiaphragm [36,39]. Patients with better diaphragm function recovery tend to show more symptom relief after PE and thoracentesis [11,28,34,36,38-41]. Post-cardiac surgery, imaging with Doppler ultrasound or fluoroscopy may reveal an elevated diaphragm and paradoxical movement, often associated with PE [36]. Elevated hemisides can result from phrenic nerve dysfunction, leading to pleural fluid accumulation due to impaired drainage [42]. This prolongs hospital stays and may cause respiratory issues requiring assisted ventilation [42]. The incidence of phrenic nerve injury after cardiac surgery varies depending on the dates of the studies examined; 46% (1993, prospective observational study, 92 patients); 21% (1998, prospective study, 63 patients); 75% (2019, prospective study, 100 patients); 38% (2020, explorative prospective observational study, 100 patients); 7,6% (retrospective observational study, 3,577 patients) [43]. The average recovery period is approximately four months [43]. Interestingly, diaphragmatic dysfunction may recur in about 26% of patients previously diagnosed with recovery [44]. Additionally, roughly 13% of patients may have pre-existing idiopathic or asymptomatic elevation of one hemiside before surgery, which could predispose them to respiratory problems and PE [45]. The diaphragm is generally not routinely assessed before or after cardiac surgery. A hemi-diaphragm following phrenic injury may appear elevated during inspiration, not only due to fluid accumulation but also because of weakened muscle strength caused by nerve impairment [46]. In cases of phrenic dysfunction with PE, the diaphragm may maintain its shape and position but often exhibits reduced contraction velocity [46]. This altered movement is more apparent on echocardiograms obtained during sitting, where diaphragmatic thickness tends to be decreased compared with normal in cases of PE and electrical dysfunction (Figure 4) [46].

**Figure 5:**
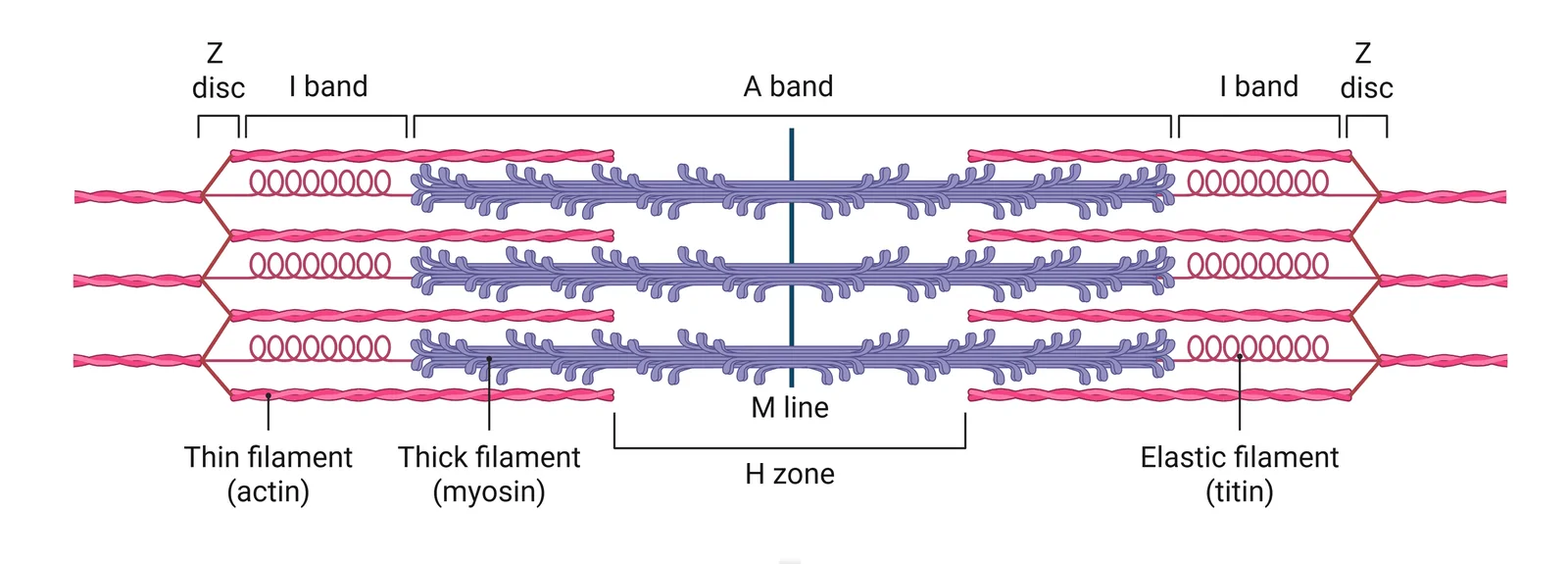
The image illustrates a schematic of the protein components of the sarcomere. In the context of cardiac surgery, the sarcomere within the diaphragmatic fibres undergoes structural and functional changes, resulting in contractile weakness. This cytotoxic environment primarily affects the red fibres [51]. The image is the property of Bordoni Bruno with a subscription to BioRender.com

Another factor that could contribute to PE and is linked to diaphragm and cardiac surgery (such as CABG and valve procedures) is altered fibre contractility, independent of phrenic nerve involvement. An observational study of 100 post-cardiac surgery patients found no iatrogenic diaphragm damage (such as paralysis or dyskinesia) or significant PE, but 38% exhibited a general reduction in range of motion, with excursion decreased by 27-40% relative to normal values [49]. This is likely due to surgical stress, intraoperative mechanical ventilation, pro-inflammatory responses, oxidative stress damaging mitochondria, and calcium-driven proteolysis and cytotoxicity within muscle fibres (Figure 5) [49-51].

Some researchers suggest this dysfunction may not be solely due to phrenic nerve injury but may also relate to decreased blood flow during internal mammary artery (IMA) involvement [47,52]. The diaphragm's rich blood supply originates from multiple arteries (phrenic, intercostal, internal thoracic), allowing relatively stable perfusion despite arterial limitations [53]. The impact of IMA grafting may not be sufficiently severe to cause diaphragmatic dysfunction. Others attribute diaphragmatic contractile deficits to thoracic drains used during surgery, which might alter muscular mechanics in the affected area [54]. Postoperative diaphragmatic weakness appears selective, primarily affecting oxidative (red) muscle fibres, with the cause remaining unclear [51]. Surgical trauma can also alter gene expression in the diaphragm, particularly by increasing pro-apoptotic markers (such as superoxide dismutase-2 and V-myc myelocytomatosis viral oncogene homolog) and inflammatory mediators (including interleukins-6, -8, and -1beta, chemokines such as C-X-C motif ligand 2 and C-C motif ligand 2, F-box proteins, and muscle-specific ubiquitin ligases) [55,56]. Multiple factors after cardiac surgery impair the diaphragm's electrical and contractile functions, thereby promoting the development of PE. Currently, no specific methods exist to prevent phrenic nerve injury or diaphragmatic damage during cardiac procedures [51,57].

Measuring diaphragmatic function

Transdiaphragmatic pressure (Pdi) induced by magnetic phrenic nerve stimulation is the invasive gold standard for assessing diaphragmatic function. Pdi is the result of gastric pressure (Pga), which reflects the force generated by diaphragmatic contraction during inspiration and the force recorded as the diaphragm descends, and of oesophageal pressure (Poe), which is the negative pleural pressure created by diaphragmatic contraction. To obtain the net contractile function, the Poe value must be subtracted from the Pga value [58]. Some catheters can measure the electrical activity of the diaphragm (diaphragmatic electromyography, EMG), providing more information on diaphragmatic and phrenic function [59]. The assessments of diaphragm contractility using this diagnostic method can vary depending on the gastric pressure already present; with gastric distension, the abdominal elastic capacity decreases, which may lead to altered values [60].

Another invasive approach to assessing diaphragmatic function is the phrenic nerve stimulation assessment [61]. Phrenic stimulation is performed via transvenous approaches in sedated patients, using electrodes placed in the jugular or subclavian vein; this approach allows the diaphragm to be activated and its functional responses to be assessed. However, this method is invasive and carries side effects, including haematoma and thrombus formation, possible infections at the catheter site, and, in rare cases, pneumothorax [62].

A study to evaluate phrenic electrical activity is performed using surface or invasive electrodes. The test evaluates the latency and amplitude of the motor response, with normal values of 0.28 mV (right hemiside) and 0.25 mV (left hemiside) for amplitude, and 8.41 ms (right hemiside) and 8.56 ms (left hemiside) for latency [63].

Other instrumental tests include fluoroscopy (during a sniff manoeuvre), radiography, magnetic resonance imaging and computed axial tomography (CT). These tests can highlight diaphragmatic anomalies in detail but may have limitations in terms of costs or the radiation the patient must undergo, or uncertainties in the evaluation between operators [64].

One of the simplest ways to measure diaphragmatic function is to use M-mode (motion mode) or B-mode (brightness mode) ultrasound. Diaphragmatic measurements (thickness and movement) are repeatable, with errors of only 0.7% [65]. Probably, B-mode ultrasound could provide more accurate measurements than M-mode during forced breathing [66].

Ultrasound is the easiest, least expensive, non-invasive, and side-effect-free method for assessing diaphragmatic function, and it can be used both in the operating room and in the outpatient clinic. Indirectly, the thickness and movement of the phrenic nerve reflect its health. An ultrasound examination performed during the post-surgery hospital stay could provide more robust indications of the need for diaphragmatic rehabilitation, and it could also provide reference values to compare with measurements derived (again from ultrasound) after the rehabilitation period. This would also facilitate the diagnostic process regarding the recovery of diaphragmatic function.

Diaphragmatic dysfunction in the presence of PE can cause atelectasis and a restrictive respiratory pattern [50]. PE adversely affects clinical parameters, which, as discussed in the article, can result in longer hospital stays and higher mortality rates. When possible, avoiding invasive procedures such as thoracentesis, which may cause significant iatrogenic complications such as pneumothorax (in up to 6% of patients) or adhesions between the pleura and surrounding tissues, is preferable, and non-invasive strategies can be employed [28,67].

Among the most commonly used tools to enhance respiratory function is continuous positive airway pressure/non-invasive mechanical ventilation (CPAP). The decision to employ it may be based on polysomnography results, in which multiple episodes of central apnea/hypopnea were identified. These episodes might increase if the patient is in lateral decubitus on the side of the body where the diaphragmatic hemi side is more affected; this serves as an indicator of diaphragmatic strain [54]. CPAP aids in the reabsorption of PE during thoracentesis within an acceptable timeframe to mitigate pleural stress (CPAP at +5 cm H2O) [68-70]. The high pressure created by CPAP facilitates the escape of PE during drainage, improving expansion, oxygenation and alveolar recruitment, and preventing fluid accumulation; CPAP counteracts the sudden intrapleural fall during mechanical or manual drainage, improving the generation of negative intrathoracic pressure [68]. Another option, not always fully exploited, is diaphragmatic muscle re-adaptation during ICU stay and hospitalization. In the presence of PE, diaphragmatic proprioception changes and dysfunction, combined with possible phrenic nerve injury and altered biomechanics, could be key factors in the worsening clinical picture [71]. When there is increased pressure on the diaphragm (such as with PE) or airway obstruction, the diaphragm increases its electrical activity, regardless of signals from the airways themselves [71]. If the diaphragm is dysfunctional (the authors use the term dysfunction to describe a diaphragm with functional difficulties), information from the phrenic receptors will impair the respiratory response, as in PE [71]. Respiratory rehabilitation re-educates the diaphragm and/or enhances recovery, thereby positively affecting breathing patterns [71]. Diaphragmatic rehabilitation, particularly IMT, improves function even in cases of post-cardiac surgery phrenic nerve injury [72,73]. IMT enhances not only inspiratory function but also expiratory function in post-extubation ICU patients [74,75]. Patients undergoing open-heart surgery can undertake IMT without side effects [76]. Considering that diaphragmatic dysfunction is an independent predictor of all-cause mortality and influences the presence of effusion, performing IMT in the ICU and post-ICU hospitalisation is crucial [45,72,77]. A recent review of 755 post-CABG and IMT patients highlighted a lower risk of developing pneumonia and atelectasis, though with limited impact on PE [78]. Why this negative outcome? When designing a training programme for a specific muscle, understanding the phenotype (fiber type present) and identifying the most appropriate stimuli and training parameters to target the fibres of interest (red or white) are essential. The review noted considerable variability in training protocols among studies, with intensities ranging from 30% to 60% of maximal inspiratory pressure (MIP), one or two sessions weekly, three to four days per week, with session durations of 5-30 minutes, involving up to three sets and 10-15 repetitions; some studies did not specify the rationale behind these choices or the parameters used for IMT [78].

Another recent review analysed 15 studies involving 815 patients undergoing cardiac surgery who used IMT before and/or after surgery. All patients used a resistive device for inspiratory muscle training, with the load set before training began. Resistance was either kept constant throughout the training period (40% of MIP) or started low and increased (from 20-40% of MIP) based on periodic reassessments of perceived exertion (Borg scale) or the subjective anaerobic threshold or was set at high levels (up to 80% of MIP) [79]. The IMT parameters used in the studies were heterogeneous. Inspiratory training ranged from three sets of 10 inhalations per session, delivered in one or two daily sessions of 20-30 minutes, to 3-8 sets of 10-15 inhalations in a single daily session, with a maximum of 30 inhalations against resistance. The duration of the training protocol ranged from 3 days to 1 year (for patients with confirmed phrenic paralysis); in most studies, IMT was applied until discharge from the inpatient ward. IMT was often used as part of a cardiac rehabilitation programme that included resistance and aerobic training [79].

Generally, studies showed improvements in lung function, inspiratory strength, and functional performance capacity; three studies reported a shorter length of hospital stay. Cardiac surgery patients who underwent IMT showed improvements in many clinical parameters. Both low and high loading (incremental resistance) with IMT were associated with benefits and no adverse events [79]. We can hypothesise that, despite the lack of homogeneity and the positive results, by using more standardised parameters, measurable clinical results could be more evident. The diaphragm facilitates changes in pleural and pulmonary pressures by stimulating the movement of excess fluids towards the lymphatic pathways; this mechanism aids pleural drainage [50]. IMT aims to improve inspiratory function and facilitate pleural drainage.

More suitable parameters to stimulate the diaphragm in the presence of pleural effusion

The phenotypic arrangement of the diaphragm in a human model shows a predominance of red, slow, aerobic fibres (type I), making up about 55%, white, fast, aerobic-anaerobic fibres (type IIA), approximately 21%, and white, fast, anaerobic fibres (type IIX), around 24% [80]. The motor units (MUs) in the diaphragm mirror this phenotypic distribution, with most being fatigue-resistant MUs for type I fibres, fatigue-resistant MUs for type IIA fibres, and fatigable fast MUs for type IIX fibres [81].

**Figure 6:**
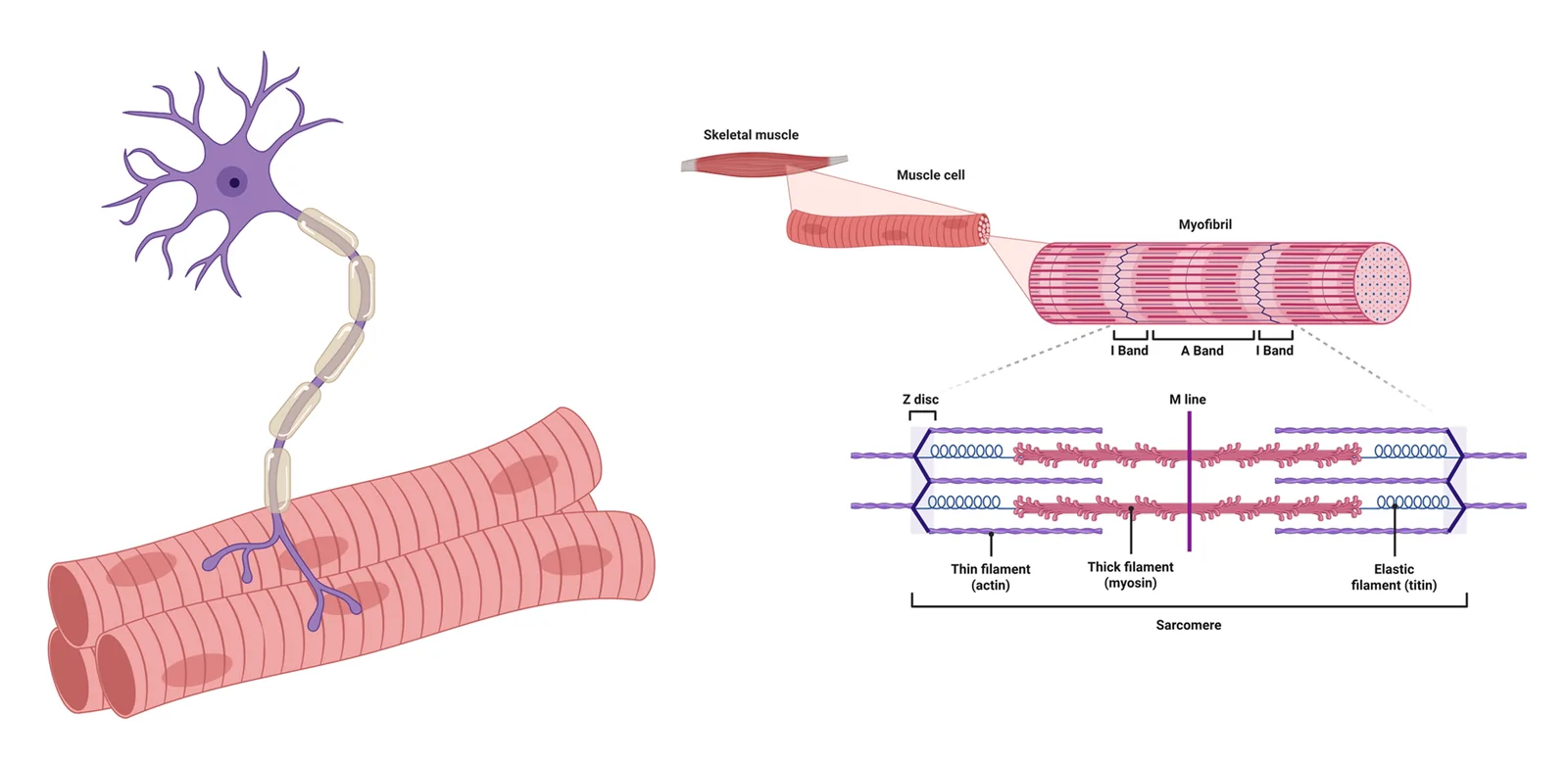
The image schematically depicts a motor unit on the left, and on the right, the organisation of the fibres up to the sarcomere and the components that determine muscle contraction. The phenotypic organisation of the diaphragm in a human model shows a predominance of red/aerobic/slow fibres (type I), with approximately 55% presence, white/aerobic-anaerobic/fast fibres (type IIA), with approximately 21%, and white/anaerobic/fast fibres (type IIX), with approximately 24% [80]. The motor units (MU) found in the diaphragm reflect this phenotypic distribution, with a majority of fatigue-resistant MUs for type I fibres, fatigue-resistant MUs for type IIA fibres, and fatigable fast MUs for type IIX fibres [81]. The image is the property of Bordoni Bruno with a subscription to BioRender.com

During restful breathing and/or during respiratory activities that exert around 40% of effort, type I fibres are primarily involved to a large extent, with type IIA fibres contributing to a lesser degree; in situations requiring greater effort, such as coughing or sneezing, it is mainly the type IIX fibres that are recruited [81,82]. Breathing that allows for an exhaustive diaphragmatic contraction (concentric) and relaxation (eccentric), as seen with aerobic fibres (unforced breathing), facilitates better oxygenation, structural adaptation, and improved functional adaptation at the neuromuscular junction [83,84]. Merely performing physical activity does not sufficiently stimulate the diaphragmatic fibres. Animal models have shown that, regardless of intensity, the diaphragm does not mirror limb adaptation [85]. It is necessary to undertake specific exercises, such as IMT (Figure 6).

Healthy subjects performing IMT with a load that challenges them during inspiration, approximately 50% of their subjective maximum inspiratory mouth pressure (PImax), for more than two minutes develop diaphragmatic fatigue, which is only compensated after about 30 minutes of rest [86]. PImax is an instrumental assessment used to evaluate inspiratory muscle strength, with values below −40 cmH2O for women and −60 cmH2O for men indicating weakness [86]. Naturally, patients with PE and post-cardiac surgery often display signs of muscular weakness [87]. How should IMT be tailored to reflect the diaphragm’s needs and function? The American College of Sports Medicine (ACSM) provides a key reference for designing muscle training programs (for further information, see [88]). According to ACSM, to stimulate the red fibres, resistance should be set at <60% of the maximum capacity (1RM), combining 15-20 repetitions or complete movements (10-15 repetitions for weaker individuals), with rest intervals of 90 seconds between sets, and a maximum of 2-3 series. The recommended frequency is to carry out training 2-4 days per week, or 2-3 days for weaker subjects [86]. Given that the fibres most involved in PE/post-cardiac surgery are the red fibres, and that excessive IMT (50% of subjective PImax or 1RM) causes contractile fatigue, diaphragmatic training in ICU should primarily target red fibres using low loads. Patients who experience pain and dizziness post-surgery cannot accurately measure maximum PImax or determine appropriate IMT resistance, as assessments may be self-limiting. It is advisable to use specific IMT devices to identify the resistance at which a patient can perform a single inspiration.

Once the patient is admitted to the ward, further tests can be performed to adjust the IMT workloads (resistances). Each forced inhalation should be followed by a rest period to ensure the next begins from a resting state, preventing hyperinflation. To further improve diaphragm function during hospitalisation or outpatient care, it would be helpful to engage the white fibres, in line with ACSM recommendations. For the white fibres (to stimulate hypertrophy/strength), the ACSM recommends a load of 70-80%, 8-12 repetitions, and approximately three minutes of rest between sets, for 2-3 sessions per week [86].

Protocol proposed by AIRC

**Figure 7:**
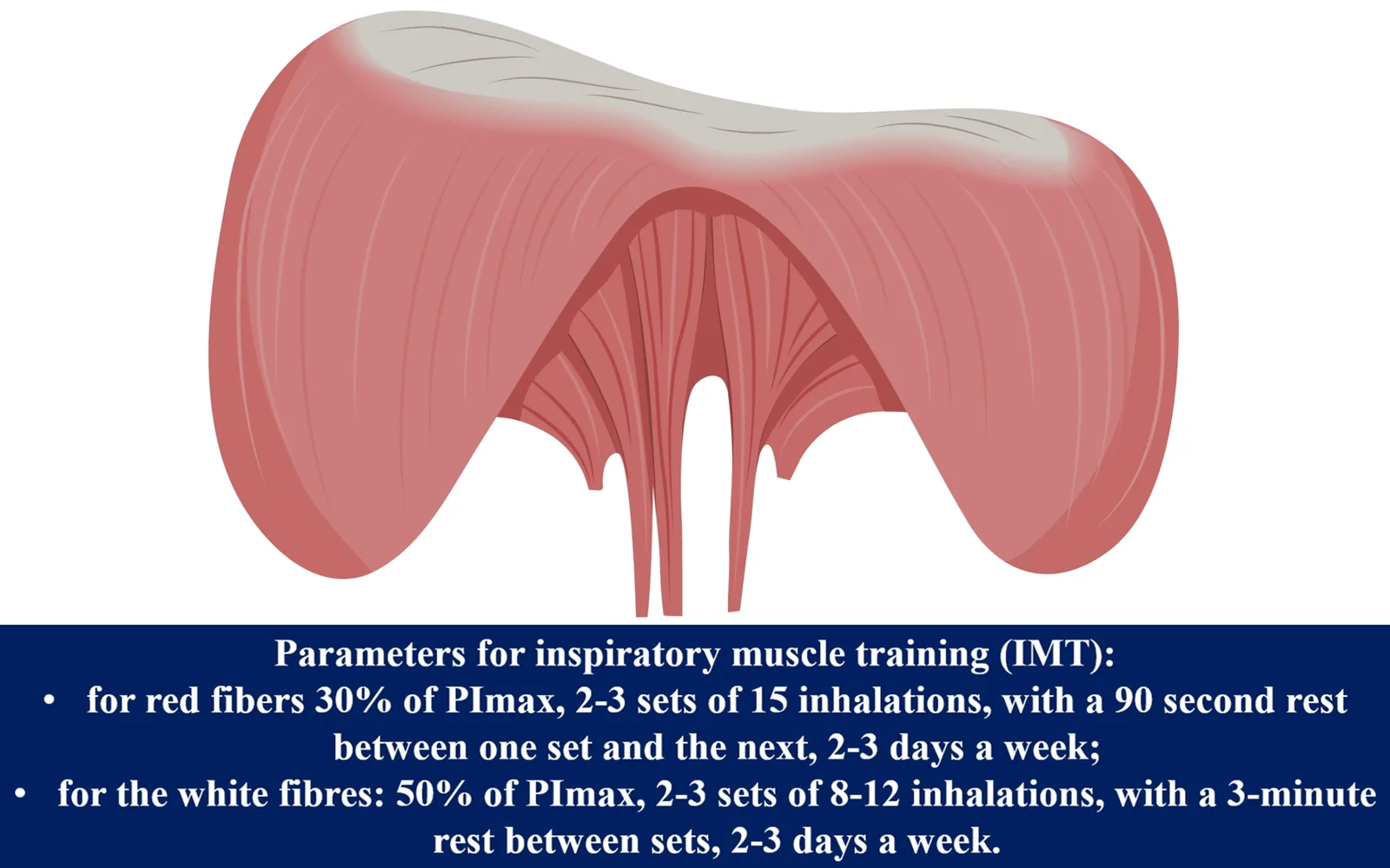
The figure outlines a working hypothesis for diaphragm training in patients in the ICU and post-cardiac surgery ward with pleural effusion. PImax can be conceptually compared to 1RM (maximum repetitions possible with a given load/resistance) [86]. A hypothesis proposed by the Italian Association of Cardiovascular Rehabilitation (AIRC) for IMT, which seeks to respect the knowledge on diaphragm fatigability and the ACSM's indications, is as follows: for red fibers 30% of PImax, 2-3 sets of 15 inhalations, with a 90 second rest between one set and the next; for the white fibres: 50% of PImax, 2-3 sets of 8-12 inhalations, with a 3-minute rest between sets, 2-3 days a week. We at AIRC suggest dividing the sessions during the week as follows: morning IMT for white fibres and afternoon IMT for red fibres (split training), with a maximum of three sessions per week. At the beginning of each week, PImax should be assessed to best adapt inspiratory resistance. The image is the property of Bordoni Bruno with a subscription to BioRender.com

PImax can be conceptually compared to 1RM (maximum repetitions possible with a given load/resistance) [86]. A hypothesis proposed by the Italian Association of Cardiovascular Rehabilitation (AIRC) for IMT, which seeks to respect the knowledge on diaphragm fatigability and the ACSM's indications, is as follows: for red fibers 30% of PImax, 2-3 sets of 15 inhalations, with a 90 second rest between one set and the next, 2-3 days a week; for the white fibres: 50% of PImax, 2-3 sets of 8-12 inhalations, with a 3-minute rest between sets, 2-3 days a week. We at AIRC suggest dividing the sessions during the week as follows: morning IMT for white fibres and afternoon IMT for red fibres (split training), with a maximum of three sessions per week. At the beginning of each week, PImax should be assessed to best adapt inspiratory resistance (Figure 7).

Our proposal is a theoretical rationale and not a standardised recommendation; further studies (prospective trials) will be necessary to validate these parameters. IMT can be used in the ICU, post-surgical wards, and outpatient settings, always respecting the patient's contingent capabilities (pain, extubation, cooperation, clinical stability). The inspiratory force (and therefore the parameters that can be used) will vary based on the patient's clinical and symptomatic conditions and their subjective recovery capacity.

Commercially available devices

What are the easy-to-use IMT devices that can be used both in a hospital setting and at home? There are several commercially available devices that provide resistance during inhalation, which forms the basis of the training. A recent study of seventy patients undergoing cardiac surgery used the POWERbreathe® and included a three-month post-cardiac surgery follow-up [89]. The group that combined standard cardiac rehabilitation with IMT and this tool demonstrated improvements not only in respiratory values such as MIP, but also in functional and muscle strength measures (six-minute walk test, Functional Independence Measure, handgrip strength and global muscle strength test, and quality of life-SF-36). POWERbreathe® improves diaphragmatic function and functional capacity in healthy elderly subjects, and it also improves ventilatory and motor function parameters in patients with chronic respiratory diseases compared with transcutaneous electrical diaphragmatic stimulation [90-92].

Another device is the SpiroTiger(®), used for chronic respiratory pathologies, based on the same principles as the previous instrument. However, there are no studies in cardiology that use this aid [92].

We do not have detailed data on phrenic nerve adaptation, but we could theoretically and indirectly infer that its electrical conduction improved through improvements in ventilatory and diaphragmatic parameters.

An advantage of using low workloads is that training is focused on the diaphragm and, to a lesser extent, on accessory muscles. In patients with unilateral diaphragmatic dysfunction, early activation of inspiratory accessory muscles (scalene, sternocleidomastoid) and increased involvement of expiratory muscles occur to compensate for diaphragmatic weakness, particularly with intense effort and high IMT workloads [93]. Excessively high IMT loads could impact the development of inspiratory accessory muscles but not the diaphragm itself [94]. IMT has been shown to improve diaphragmatic function in patients undergoing median sternotomy for cardiac surgery [72]. We conclude by emphasising that IMT should not stop upon discharge from hospital but should continue for several months (about four), respecting the diaphragm's recovery timeline and preventing new effusions. We do not yet know how patients respond to the IMT protocol outlined here to accelerate diaphragmatic resorption and functional recovery. Further research is necessary to better understand optimal diaphragm stimulation parameters in PE and to determine if these training intensities can lead to meaningful clinical improvements.

Contraindications and precautions for IMT post-cardiac surgery

IMT does not show side effects when the clinical picture is stable in post-cardiac surgery patients [95]. Caution should be exercised when using IMT in patients who are not clinically stable: acute respiratory infections; uncontrolled hypertension or hypotension; non-cooperation due to cognitive impairment; unevaluated or unoperated aneurysms; spontaneous pneumothorax; an abdominal wound from recent surgery; severe pain reported by the patient; evidence of haemoptysis of unknown aetiology; acute events (such as haemorrhages, fractures); bullous emphysema; severe nausea and/or vomiting; haemodynamic instability; diastatic sternal wound; frequent ventricular arrhythmias or acute atrial fibrillation [96,97]. To confirm the absence of adverse side effects, a recent review of IMT and cardiac surgery (excluding PE) reaffirmed the safety of inspiratory training [98].

## Conclusions

This article examined possible causes and risk factors for PE following cardiac surgery and updated the definition of this clinical complication. It reviewed how the diaphragm's function and structure adapt in the presence of PE, aiming to highlight the significance of inspiration in potentially resolving the pulmonary problem and achieving faster symptom relief. It offered suggestions for designing diaphragm training programmes based on muscle physiology and emphasised the importance of prioritising specific metabolic adaptations caused by effusion. Further research should explore common strategies for implementing a diaphragm training programme in the presence of PE to achieve more consistent outcomes.
